# University of Louisville

**Published:** 1847-04

**Authors:** 


### UNIVERSITY OF LOUISVILLE.

The Commencement in the Medical Department of the University of Louisville was held Saturday evening, the 6th of March, when seventy-five gentlemen, who had passed satisfactory examinations before the faculty, and otherwise complied with the requirements of the institution, received the degree of Doctor of Medicine. James C. Harris, M.D., of Wetumpka, Alabama, a graduate of Transylvania University; David A. Post, M.D., of Orangeburg, Ky., a graduate of the Medical Department of Western Reserve College, Ohio; and Thos. J. Todd, M.D., of St. Joseph, Mo., a graduate of Transylvania University, were admitted to the Ad-eundem Degree in this University. The Honorary Degree of Doctor of Medicine was conferred upon Dr. Cyprian P. Mattingly, of Bardstown, Ky., Dr. William J. Johnson, of Fort Gaines, Georgia, and Dr. Elijah Newland, of Salem, Indiana.

An appropriate and instructive valedictory address was delivered to the graduates by Prof. Short, in presence of a brilliant assembly which had met to witness the interesting ceremonies of the occasion. The professor lashed some of the professional vices of the day with merited severity, while he exhorted the graduates to a life of diligent study, by which alone they could hope to win an enduring name as learned and skilful physicians.

